# A Nonseminomatous Germ Cell Tumor Presenting as a Mixed Cryoglobulinemic Vasculitis

**DOI:** 10.1155/2022/3326761

**Published:** 2022-12-23

**Authors:** Gabriel Cojuc-Konigsberg, Isabel Natera-Comte, Blanca E. López Graciano, Luis Gerardo Mosqueda López, José Alonso Ávila-Rojo, Braulio Martínez, Juan C. Ramírez-Sandoval

**Affiliations:** Instituto Nacional de Ciencias Médicas y Nutrición Salvador Zubirán, Mexico

## Abstract

**Background:**

Mixed cryoglobulinemia syndrome (MCS) is a rare entity with a variety of causes but has not been associated with testicular germ cell tumors. We present here a case of a patient with a nonseminomatous germ cell tumor (NSGCT) presenting as a type III mixed cryoglobulinemic vasculitis. *Case Presentation*. A 58-year-old male exhibited typical clinical features of vasculitis, including weakness, fatigue, palpable purpura, multiple mononeuropathy, and a low C4 level. An MCS diagnosis was confirmed by the presence of cryoglobulins (6%) with polyclonal IgM and IgG components and biopsy proven leukocytoclastic vasculitis. Concomitantly, a stage IIIC (TxNxM1bS1) germ tumor with marked elevation of serum beta-human chorionic gonadotropin (2764 mUI/mL) was diagnosed. An aggressive treatment was needed, including methylprednisolone pulses, plasmapheresis, rituximab, followed by orchiectomy, and chemotherapy (bleomycin/etoposide/cisplatin). After tumor resection and treatment, cryoglobulins decrease to 0%, suggesting a paraneoplastic origin of the vasculitis.

**Conclusion:**

To the best of our knowledge, this is the first case of MCS possibly attributable to a NSGCT. This case further elaborates on the presentation of mixed cryoglobulinemia vasculitis and adds to the published literature on the topic.

## 1. Introduction

Mixed cryoglobulinemia syndrome (MCS) is a rare condition (1 : 100,000) characterized by a systemic inflammatory syndrome caused by the deposition of antigen-antibody complexes in capillaries and small arterioles caused by either type II or type III cryoglobulins (cryoglobulins that contain more than one immunoglobulin component such as IgM, rheumatoid factor, and polyclonal IgG) [1]. MCS is commonly secondary to other diseases such as hepatitis C, autoimmune conditions, and lymphoproliferative disorders like diffuse large B cell lymphoma and non-Hodgkin lymphoma [2]. To our knowledge, we describe the first report of a subject with paraneoplastic type III cryoglobulinemic vasculitis as the initial presentation of a germ cell tumor. This work has been reported in line with the CARE criteria [3]. We believe that this case further elaborates on the presentation of mixed cryoglobulinemia vasculitis and adds to the published literature on the topic.

## 2. Case Presentation

**Figure 1:**
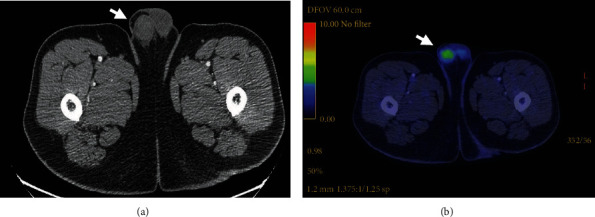
FDG-PET/CT scanning revealed a testicular mass, with maximal SUV of 6.9 (arrow): (a) transverse image and (b) CT of the testicular mass.

**Figure 2:**
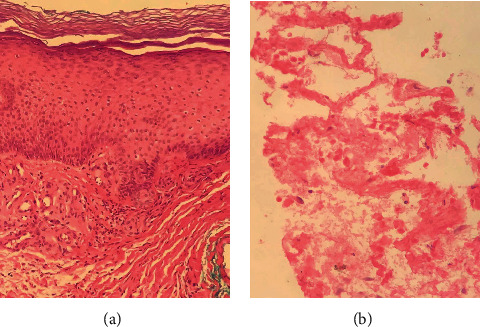
Skin (a) and lung biopsies (b) performed three months before admission (400x, H&E).

A 58-year-old male from Mexico City with a history of orchiopexy for cryptorchidism at 14 years of age had been well until approximately 7 months before admission to this hospital when weakness, fatigue, and erythematous macules, edema, and paresthesia of the lower extremities developed. He had previously been diagnosed with obesity (body mass index of 38 kg/m^2^), prediabetes, mixed dyslipidemia, obstructive sleep apnea, and a multinodular goiter. His history revealed a smoking index of 19 pack-years and physical inactivity. The patient was initially assessed by an independent physician who prescribed ruscus extracts, hesperidin, and ascorbic acid. Five months before admission, the symptoms were persistent, with the appearance of new skin ulcers, tensional headache, intermittent chest pain, myalgia, and worsening of dysesthesias and pain in the legs. Three months before admission to this hospital, the patient was referred to a specialist. A PET-CT scan exhibited multiple hypermetabolic areas described as pulmonary nodules and retroperitoneal adenopathies, compatible with metastatic lesions (Figure 1), as well as an enlargement in the right testicle, which was not clinically manifest. A skin biopsy was performed, resulting in leukocytoclastic vasculitis and intimal hyperplasia (Figure 2(a)). A lung biopsy showed alveolar hemorrhage with fibrinoid necrosis and transmural infiltrates, suggestive of vasculitis (Figure 2(b)). Laboratory tests showed a high lactate dehydrogenase (LDH) concentration, low levels of C4 (9.5 mg/dL, reference 19-52 mg/dL), and normal C3 levels. Testing for hepatitis C virus and others such as antinuclear antibodies, anti-double-stranded DNA, or ANCA were negative (Table 1). An initial diagnosis of small vessel vasculitis was made, yet a specific etiology was not identified. The patient received methylprednisolone 500 mg at weekly intervals for 3 doses, prednisone 60 mg qd, and mycophenolate mofetil 500 mg bid. The subject was referred to our institution for further assessment.

On admission to this hospital, the patient reported improvement of the skin lesions after treatment with steroids but persisted with significant neuropathic pain. Upon physical examination, livedo reticularis, retiform purpura, and multiple ulcers were found. Results of the cryoglobulin assay showed a cryocrit of 6% (reference < 1%), and immunofixation of the cryoprecipitate revealed polyclonal IgM and IgG components. The serum viscosity was 1.7 (expressed as a value relative to water). A slightly low C4 level (22 mg/dL, reference 19-52 mg/dL) was detected. Electrophoresis phase of immunofixation showed polyclonal bands in serum, yet the fixation phase was negative for monoclonal protein in neither serum nor urine. Quantitative free light chain measurements were not performed. Subnephrotic proteinuria of 311 mg/day, low 25-hydroxyvitamin D, leukocytosis, and polycythemia were other relevant findings. Additionally, patient had high levels of (LDH) (272 U/L, reference 140-271 U/L) and beta-human chorionic gonadotropin (beta-hGC) (2764 mU/mL, reference 0.5-2.67 mUI/mL, confirmed by serial dilution up to 1 : 100, immunoassay system, Beckman Coulter). Other biomarkers including carcinoembryonic antigen, alpha-fetoprotein, and CA 19-9 were normal (Table 1). Because of the peripheral nerve manifestations, a nerve conduction velocity test was performed, detecting severe, sensorimotor asymmetrical axonal damage, indicative of multiple mononeuropathy. Based on the clinical, laboratory, and histopathological findings, a diagnosis of type III or mixed cryoglobulinemic vasculitis was made, initiating treatment with high-dose corticosteroids (prednisone 60 mg/day) and rituximab (two 1 g induction doses), as well as symptomatic measures. Many possible etiologies were excluded with the pertinent assessment, including neoplastic, infectious, and rheumatologic diseases.

**Figure 3:**
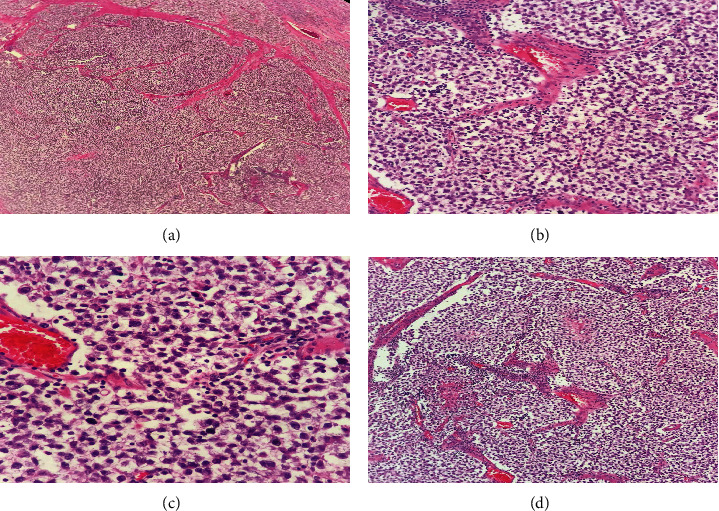
(a) Germ cell tumor neoplasm with solid and lobular pattern and fibrous septae (40x, H&E). (b) Tumor cells are large with pale cytoplasm. (c) The nuclei are round with one prominent central nucleoli. (d) A prominent lymphocytic infiltrate is present (400x, H&E).

Upon further examination, a petrous enlargement of the right testicle was found. The patient was referred to the urology department, where a scrotal ultrasound revealed a mass. A radical right orchiectomy was performed, with a final pathology report of a stage IIIC (TxNxM1bS1) germ cell tumor with beta-hGC levels > 1,000 IU/L consistently with the diagnosis of nonseminomatous germ cell tumor (NSGCT) (Figure 3). Chemotherapy with BEP (bleomycin, etoposide, and cisplatin) as adjuvant chemotherapy regimen was initiated [4].

Six months after chemotherapy, follow-up studies were requested, showing that after tumor resection and treatment cryoglobulins were now at 0%, suggesting a paraneoplastic origin of the vasculitis. Regarding the clinical manifestations of cryoglobulinemia, some sequelae remained, such as chronic ulcers and predominantly sensitive peripheral nerve damage, which were treated with 3 sessions of plasmapheresis and collagenase-chloramphenicol ointment, respectively. In the follow-up, a relapse of NSGCT was diagnosed by a sudden increase in beta-hGC concentrations with distal multiple metastases resistant to initial chemotherapy. Concomitantly, a 2% increase in cryoglobulin levels was found.

## 3. Discussion

In this case, we describe a patient with a metastatic NSGCT who presented as initial clinical symptoms weakness, fatigue, palpable purpura, multiple mononeuropathy, and hypocomplementemia with exclusively low C4 level. MCS diagnosis was confirmed by the presence of cryoglobulins in serum and findings of vasculitis in skin and lung biopsies. Although it was not possible to perform cryoglobulin determination in the biopsies, we performed a full rheumatologic, hematologic malignancy, and concomitant infection workup to exclude secondary causes of MCS. After excluding other medical reasons, we confidently made a diagnosis of MCS secondary to a stage IIIC NSGCT. The patient's response to treatment further supports that this was an immune-related phenomenon. The criteria employed in this case to support the association were (1) definite diagnosis of NSGCT, (2) MCS occurrence concurrent with NSGCT diagnosis, (3) exclusion of other causes of MCS, and (4) the changes of cryoglobulin levels after therapy initiation, including chemotherapy and orchiectomy. These criteria are essentially the same as those established for paraneoplastic neurological syndromes [5].

MCS is the result of immune complex deposition in vessel walls, typically seen in association with hepatitis C virus infection. MCS is less commonly seen with systemic lupus erythematosus, Sjögren's syndrome, or as a manifestation of immune-mediated paraneoplastic syndromes, commonly lymphomas [6–8]. Nevertheless, hematological autoimmune reactions associated with solid tumors are uncommon, and the stimulus for autoantibody production is uncertain. Malignant solid cancers are associated with multiple paraneoplastic syndromes, directly linked to local or metastatic tumor growth, nutritional deficiencies, infections, metabolic disorders, or ectopic production of biological substances [9]. Autoimmune paraneoplastic syndromes related to solid tumors are occasional; the most frequent are autoimmune hemolytic anemia and autoimmune thrombocytopenia [8]. The occurrence of MCS in solid tumors is very rare, and their association is only supported by case reports. Only a few cases of this association have been described in atrial myxoma [10], Kaposi's sarcoma [11], mycosis fungoides [12], nasopharyngeal carcinoma [13], and lung cancer [14].

Among patients with NSGCT, paraneoplastic syndromes are uncommon. NSGCT has been linked to the production of specific serum proteins, especially beta-hCG which is associated with hyperthyroidism [15]. In 10% to 40% of patients with NSGCT, an elevated serum beta-hCG is observed and correlates with metastatic status and increased tumor burden [16]. Nevertheless, beta-hCG-induced hyperthyroidism is rare and has been reported at very high beta-hCG concentrations, usually greater than 50,000 IU/L [15, 17]. Another paraneoplastic syndrome described in patients with NSGCT and elevated beta-hCG has been a tendency to pulmonary bleeding, hypoxia, and acute respiratory distress syndrome, usually after chemotherapy administration [18]. Hypercalcemia due to PTH-like protein or 1 alpha-hydroxylase activity in tumoral germ cells has been described [19]. In 1977, Lundberg and Mitchell [20] described a patient with a germinal tumor (pure seminoma), severe warm autoimmune hemolytic anemia caused by IgG antibodies, and type I cryoglobulins (monoclonal IgM-kappa) without data of the classical cryoglobulinemia syndrome. We are not aware of any other reports of NSGCT associated with the presence of cryoglobulins. The rarity of type III MCS associated with NSGCT and the lack of previous reports justify the reporting of this case. We highlight the importance of performing a thorough clinical examination and an extensive clinical approach in search of neoplasia in those cases of MCS where a clear etiology is elusive.

## Figures and Tables

**Table 1:** Laboratory values.

Variable	3 months before evaluation	On hospital admission	4 months after admission	7 months after admission	Reference range
Hemoglobin (g/dL)	15	18.2	17.2	16.7	13-1-18.1
White cell count (per mm^3^)	6.2	4.1	11	10.5	3.9-10.1
Platelet count (per mm^3^)	174	172	158	170	147-402
Creatinine (mg/dL)	1.1	0.94	1.08	1.08	
C3 complement (mg/dL)	122	145.8	182		87-200
C4 complement (mg/dL)	9.5	16.7	22	32	19-52
Rheumatoid factor (UI/mL)	17		7.9	<10	0-14
Cryoglobulins (%)	—	6%	0%	2%	≤1
Hepatitis B surface antigen		Negative			Negative
Hepatitis C antibody		Negative			Negative
Erythrocyte sedimentation rate (mm/Hr)		4			2-30
C-reactive protein (mg/L)	98	33	12		<10
LDH (U/L)		269	182	210	140-271
MPO (U/mL)		1.3	1.2	—	≤2.0
PR3 (U/mL)		2	2	—	≤2.0
ANA		Negative			≤1 : 20
ANCA		1 : 20			≤1 : 20
Anti-SM		<1			<1
Anti-dsDNA		<1			<1
Anti-SSA (U/mL)		6.4			≤9.1
Anti-SSB (U/mL)		5.3			≤7.0
Beta-hCG (mUI/mL)	933	2764	5764	7825	0.5-2.67
Alpha-fetoprotein (ng/mL)	1.28	1.36	1.96	1.47	0-9
CA 19-9	8				<34
CEA (ng/mL)	1.43				<9
Proteinuria (mg/day)	606	311			<150

ANA: antinuclear antibody by indirect immunofluorescence; ANCA: antineutrophil cytoplasmic antibodies; Anti-dsDNA: antidouble stranded DNA; CA 19-9: carbohydrate antigen 19-9; CEA: carcinoembryonic antigen; beta-hCG: beta-human chorionic gonadotropin; LDH: lactate dehydrogenase; PR3: proteinase 3; MPO: myeloperoxidase.

## Data Availability

The data that support the findings of this study are available from the corresponding author, JCRS, upon reasonable request.
